# Ethanol photocatalysis on rutile TiO_2_(110): the role of defects and water

**DOI:** 10.1039/c5cp03550c

**Published:** 2015-07-30

**Authors:** Constantin A. Walenta, Sebastian L. Kollmannsberger, Josef Kiermaier, Andreas Winbauer, Martin Tschurl, Ueli Heiz

**Affiliations:** a Chair of Physical Chemistry, Department of Chemistry and Catalysis Research Center, Technische Universität München Lichtenbergstrasse 4 85748 Garching Germany ulrich.heiz@mytum.de +49 (0)89 289 13389 +49 (0)89 289 13391

## Abstract

In this work we present a stoichiometric reaction mechanism for the photocatalytic ethanol oxidation on TiO_2_(110). The reaction products are analyzed either under reaction conditions or after irradiation at lower temperatures. Water is identified as a quantitative by-product, which resides in a defect site. These water molecules cause a blocking of the defect sites which results in poisoning of the catalyst. By different preparation techniques of the TiO_2_(110) surface, the role of surface defects is further elucidated and the role of molecular oxygen is investigated. Based on the investigation, a complete photochemical reaction mechanism is given, which provides insights into general photon driven oxidation mechanisms on TiO_2_.

## Abstract 2

In this work we present a stoichiometric reaction mechanism for the photocatalytic ethanol oxidation on TiO_2_(110).

## Graphical abstract

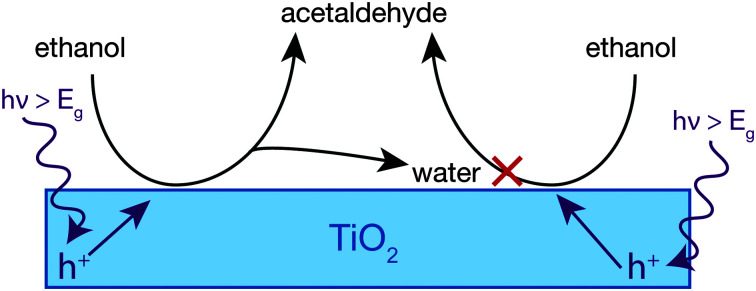

## Introduction

The thermodynamically stable rutile TiO_2_(110) surface is among the most studied oxide model systems in surface science. Since the discovery of its photocatalytic activity, TiO_2_ has become the most explored heterogeneous photocatalyst to date.^1–5^ While photocatalytic mechanisms of simple model reactions (as the CO-oxidation, or the O_2_-photon stimulated desorption [PSD]) on the surface are well understood,^6–9^ the photochemical mechanisms of small organic molecules on bare TiO_2_(110) remain unclear. For almost any reaction not even all reaction products have so far been identified.^10–17^ The understanding of the exact photocatalytic processes for such reactions will, however, be highly beneficial, since a fundamental understanding will allow for improvement of titania-based photocatalysts, in general. This is also the reason why the photochemistry of such systems has attracted considerable attention within the last few years.^5,18,19^ In this regard, ethanol is in particular of significant interest, because this molecule serves as a precursor for biomass fuels, due to its C–C-bond.^20^ Furthermore, its functional group makes ethanol a potential precursor for the green photocatalytic hydrogen production, especially since alcohols can be obtained as a renewable feedstock from biomass.^21–23^ Therefore, the elucidation of the ethanol chemistry on TiO_2_ represents an important step towards the understanding of the H_2_-production from renewable sources with light on metal-semiconductor hybrid materials.

Considering the reaction pathway, it is known that alcohols generally undergo a hole-mediated oxidation process on a rutile TiO_2_(110) surface.^5,8,10,11,16^ Idriss and coworkers observed the photoreaction of ethanol to acetaldehyde with a strong dependency on the oxygen pressure on an oxygen covered surface. In a more recent study, however, Yang and co-workers have reported a photo-oxidation of ethanol under exclusion of oxygen, focussing on its photocatalyzed dissociation.^24^ For adsorbed propanol, analogous production of propanal was observed and a similar dependence on the O_2_ concentration was found as well.^10,25^ The group of Henderson reported the oxidation of methanol to formaldehyde on a defect-rich crystal surface in the absence of any O_2_ and determined the surface-bound methoxy as the active reactant.^16,26^ Consecutive photocatalyzed coupling reactions have also been reported in the literature.^27,28^

All studies up to date have only investigated the main photo-product and postulated mechanisms based on plausible assumptions. Thus, the stoichiometric reaction equations have been made without the analysis of any by-products and unknown charge states. For the total reaction pathway, the role of defects in the reaction still remains unclear, especially when oxygen is added as a reactant. In this work stoichiometric mechanisms for the photochemistry of ethanol on rutile TiO_2_ are presented. The mechanisms unravel the role of oxygen and surface defects and are supported by studies based on different preparation conditions for the titania crystal and on the analysis of water as one of the main products of the photoreaction.

## Experimental

**Fig. 1:**
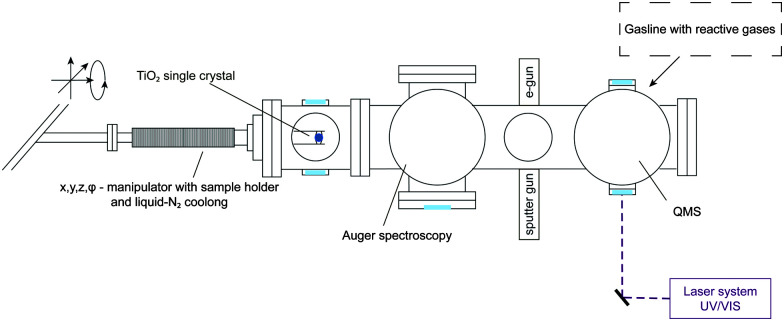
Overview of the apparatus including methods for surface characterization and preparation as well as product analysis.

All experiments were carried out in a home built ultra-high vacuum setup as seen in Fig. 1 with a base pressure of 8.0 × 10^−11^ mbar. Briefly, it consists of a liquid-N_2_ cooled, *x*, *y*, *z*, *ϕ*-manipulator (VAB Vakuum GmbH), an Auger Spectrometer (CMA 100, Omicron Nanotechnology GmbH), a sputter gun (IQE 11/35, SPECS GmbH), a QMS (QMA 430, Pfeiffer Vacuum GmbH), leak valves (Pfeiffer Vacuum GmbH), a home-built gasline (base pressure of 5.0 × 10^−9^ mbar) and an e-gun of in-house design.

The rutile TiO_2_(110) crystal was purchased from Surface-net GmbH and is of cylindrical shape with a diameter of 10 mm and a thickness of 2 mm. At two sides of the crystal symmetrical grooves were cut for mounting the crystal on a 1 mm thick tantalum plate. The interface between the metal and the single crystal is covered with a thin (0.025 mm) gold foil to ensure good thermal conductivity. The heating of the crystal was performed indirectly *via* resistive heating of two tungsten wires (0.38 mm in diameter), which were fixed on the sides of the tantalum holder. For the photochemical measurement, the sample holder was cooled by contact to a reservoir of liquid nitrogen to achieve temperatures of around 100 K. The temperature of the crystal was controlled by the calibrated readout of a twisted type-C thermocouple,^29^ which was inserted into a hole located on the side of the rutile single crystal without any adhesive. Crystal cleaning was done by repeated cycles of Ar^+^-sputtering, oxygen annealing and vacuum annealing and confirmed by AES. With this procedure a reduced, dark blue, conductive crystal was obtained which has a constant surface defect density of usually 10 to 15%.^3,30,31^

Ethanol (absolute, HPLC grade, ≥99.8%, Sigma-Aldrich) was purified by pump-thaw cycles and flushing cycles of the gasline prior to use. Purity was confirmed by chamber backfilling and analysis with the QMS. The ethanol dosage was performed at a crystal temperature below 150 K and TPD experiments were conducted by heating the crystal with 1.2 K s^−1^ to 700 K. For the photochemical measurements a Nd:YAG-pumped (Spectra GCR 4, ∼10 ns pulse length) dye laser (Lambda Physics) with a wavelength of 266.5 nm is used to excite electron–hole pairs. The laser spot lights the entire single crystal plane and the intensity was chosen with a pulse energy of 600 μJ per pulse, so no laser induced thermal heating effects were observed. Mass signals in the QMS of thermal and photochemically desorbing species were identified by cracking pattern analysis and were corrected by the different ionization sensitivities.

## Results

**Fig. 2:**
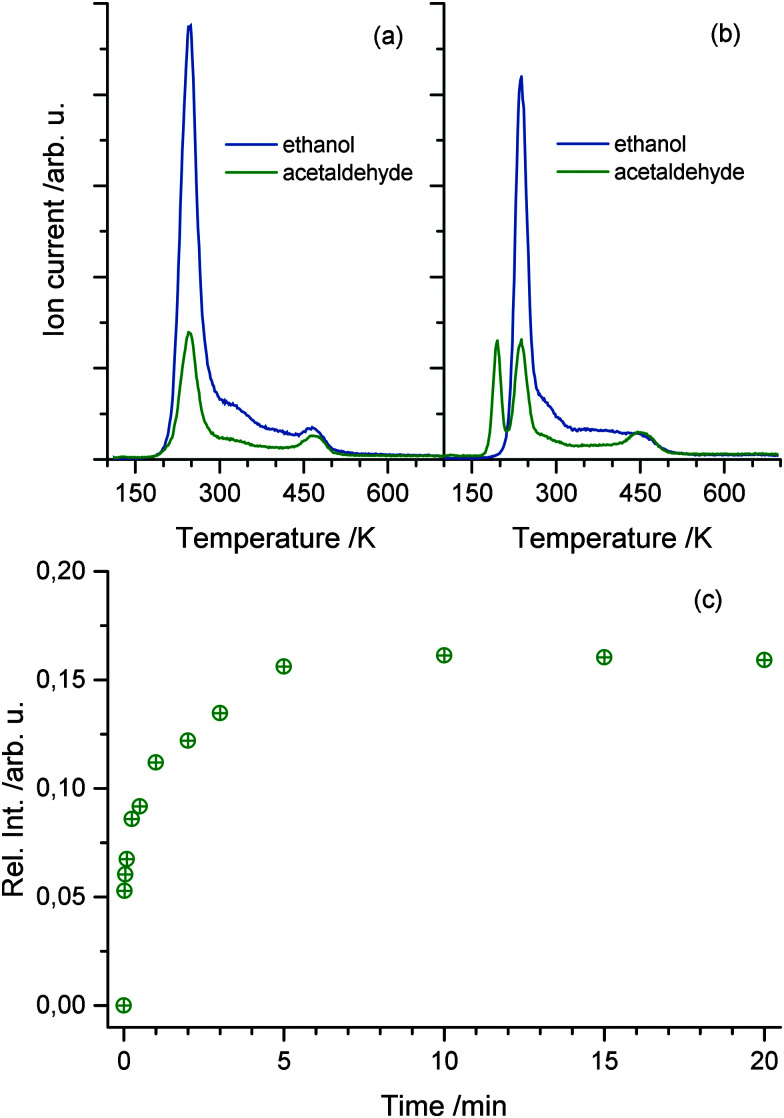
(a) The thermal desorption mass signal of acetaldehyde and ethanol without photoexcitation. Note that the trace assigned to acetaldehyde is a fragment of either ethanol or acetaldehyde. Due to the constant ratio of both traces, the origin of the first peak is assigned to ethanol desorption, while acetaldehyde production only occurs at temperatures above 400 K. (b) The thermal desorption mass signal of acetaldehyde and ethanol after 10 min of UV excitation at 110 K. The consecutive TPD run shows another feature at 195 K, resulting from molecular acetaldehyde. (c) The integral over acetaldehyde production normalized to the overall ethanol dosage. The signal shows a rapid rise in acetaldehyde production within 1 s of photoexcitation and a saturation of acetaldehyde is found after 5 min of photoexcitation.

For the elucidation of the mechanisms for the photocatalytic ethanol oxidation reaction, different desorption experiments were performed. The temperature programmed desorption (TPD) spectrum of 1 L of pure ethanol in the absence of UV light is shown in Fig. 2a. From the fragmentation pattern analysis, the traces were assigned to species. The trace (*m/z* = 31) corresponds to the main fragment of ethanol and (*m/z* = 29) to that of acetaldehyde. For the latter, it has to be taken into account that this mass appears in the main fragmentation pattern of ethanol as well. The TPD spectra of the pure alcohol have already been extensively discussed in the literature.^32–34^ In general agreement, it is found that the majority of ethanol molecules desorb at temperatures below 300 K and, besides other features, another distinct peak above 400 K occurs. The trace representing acetaldehyde follows the same trend, but is proportionally lower in intensity. This trend only deviates at higher temperatures indicating that the signal only originates from the fragmentation pattern of ethanol with the exception of temperatures above 400 K, where also ethene is produced from dehydration.^32–34^

Upon irradiation of photons with energies above the band gap, another feature at 195 K appears in the spectrum depicted in Fig. 2b. This peak can solely be assigned to acetaldehyde, because no desorption of ethanol is observed at this temperature. This clearly shows that in the absence of O_2_, photo-excitation at 110 K results in the accumulation of acetaldehyde at the surface. In agreement with the literature for highly covered crystal surfaces, the desorption of acetaldehyde occurs at lower temperature due to the repulsive interactions of the molecule with ethanol and other surface species.^13,24^ In contrast to the study of Idriss and coworkers performed at 300 K^12^ and in agreement with Yang and co-workers,^24^ oxygen dosing is not a prerequisite for a substantial yield in the ethanol photooxidation at lower temperatures. The amount of acetaldehyde produced is strongly dependent on the illumination time. Fig. 2c shows the integrated mass signal of acetaldehyde trace normalized to the ethanol coverage *versus* illumination time at 110 K. A rapid rise with the photoexcitation is observed, which results in saturation at about 15% with respect to ethanol after 5 min.

**Fig. 3:**
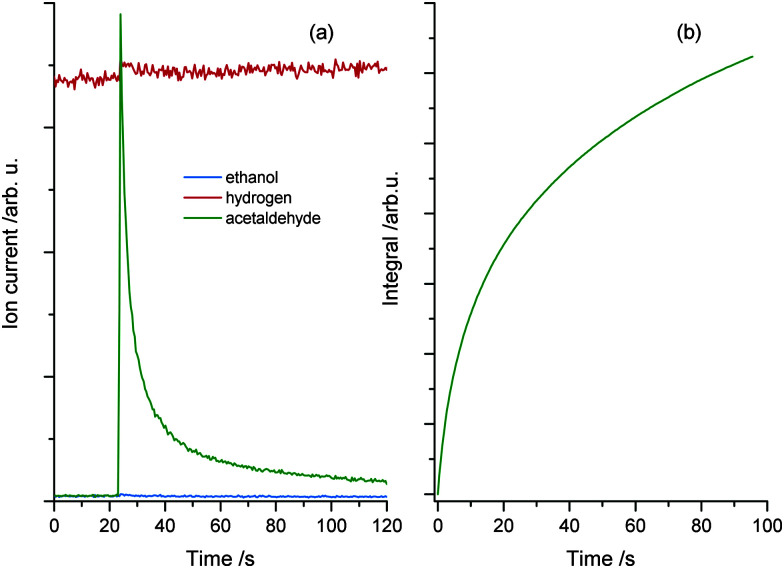
(a) Isothermal photoreaction at 222 K. Prior to reaction, 1 L ethanol is dosed at a temperature of 120 K. UV excitation starts at 23 seconds and an immediate increase in acetaldehyde production is observed, while the ethanol trace remains unchanged. Note that the slight change in the molecular hydrogen trace is attributed to cracking in the QMS rather than to the production of H_2_. Part (b) shows the integrated mass signal of figure (a), which shows the same trend as Fig. 2(c).

Since the desorption of acetaldehyde takes place at lower temperatures than that of ethanol, the photochemical reaction can be monitored *in situ*. Fig. 3a demonstrates the formation and the direct desorption of the aldehyde under UV illumination at 222 K, which is well below the desorption temperature of the alcohol. Since this method enables the recording of any product that leaves the surface at this temperature, the H_2_ mass can also be monitored. However, only a small change in the signal with UV excitation is seen, which is rather attributed to cracking in the QMS than to the production of molecular hydrogen during reaction. Furthermore, no ethanol desorption is observed as is expected at this temperature. As seen in Fig. 3b the integral of the photoproduct matches the amount of acetaldehyde produced at cold temperatures (Fig. 2c), showing that the reaction is charge carrier driven and desorption only occurs *via* a thermal process. After another dosage of ethanol at lower temperatures and subsequent irradiation at 222 K, a strong decrease in the acetaldehyde production is observed. The photochemical yield gets even lower for all successive dosage cycles, which hints to a deactivation mechanism of the catalyst. However, after ramping up the temperature to about 500 K, the activity of the catalyst can be completely restored.

**Fig. 4:**
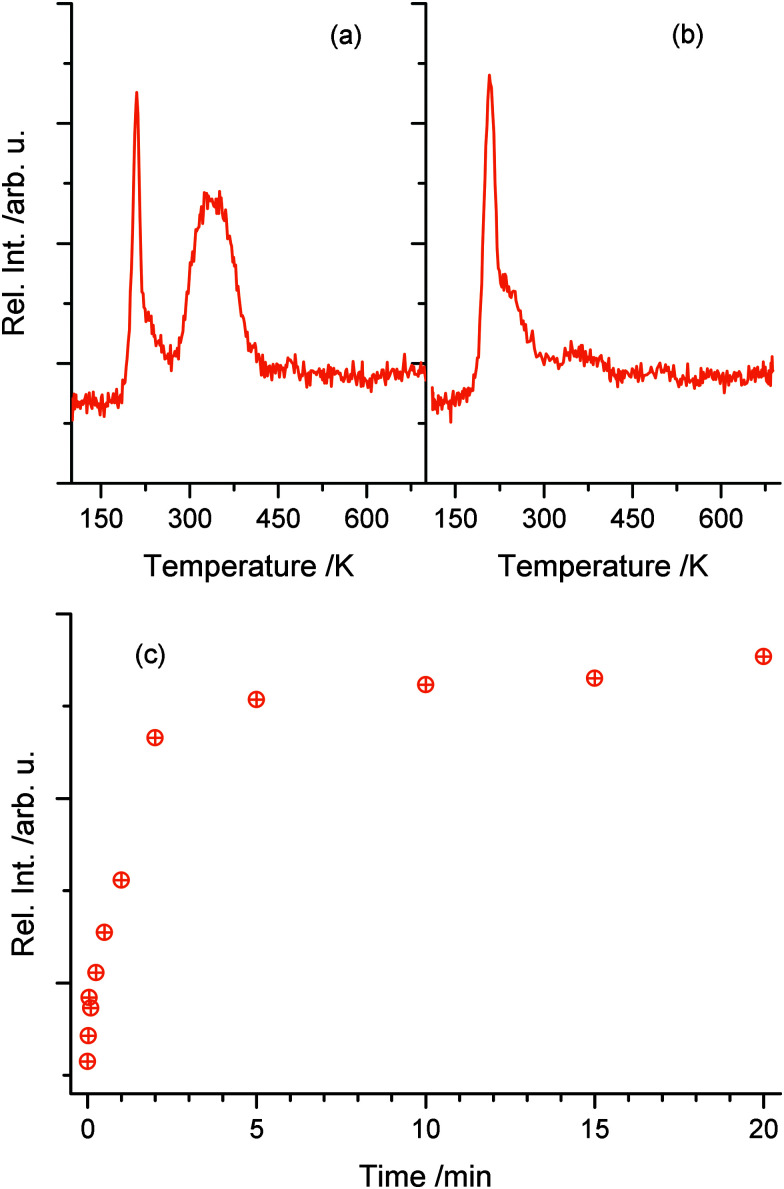
The upper parts ((a) and (b)) show the water mass traces in TPD experiments of 1 L ethanol after 5 min of photoexcitation (a) and 5 min of waiting without photoexcitation (b). In both cases, a sharp feature of water at 210 K is observed. However, only with UV illumination, a high temperature feature between 290 K and 420 K occurs, which demonstrates that water is formed during the photoreaction. Part (c) shows the integral of high temperature water in dependence of the photoexcitation time probed by TPD. Similar to Fig. 2(c), a rapid rise occurs with a saturation at a UV-excitation time of 5 min.

While no significant H_2_ production is found in the photoreaction, it is observed that water molecules (*m/z* = 18) are leaving the surface in the TPD experiments in good agreement to Yang and co-workers.^24^ However, such water molecules may either originate from the photochemical reaction or the co-adsorption of residual water molecules from ethanol dosage. Thus, Fig. 4 shows a comparison of the H_2_O trace of 1 L ethanol after 5 min UV illumination (a) and after the same time in the dark (b), to enable the discrimination between water formed by the photoreaction and water from co-adsorption. In both cases, an identical feature at 210 K is found, which can be attributed to H_2_O desorbing from O-bridge atoms of the semiconductor. However, in the case of the photoexcitation (Fig. 4a) another distinct feature between 280 K and 400 K occurs, which is assigned to water coordinated on Ti^4+^-sites in the TiO_2_ lattice.^34^Fig. 4c demonstrates that the integral of the peak from these Ti sites *versus* time shows a similar behavior to the acetaldehyde formation (Fig. 2c). Thus, the production of water in this defect sites is clearly associated with the photochemical process and is identified as a quantitative by-product of the photoreaction.

**Fig. 5:**
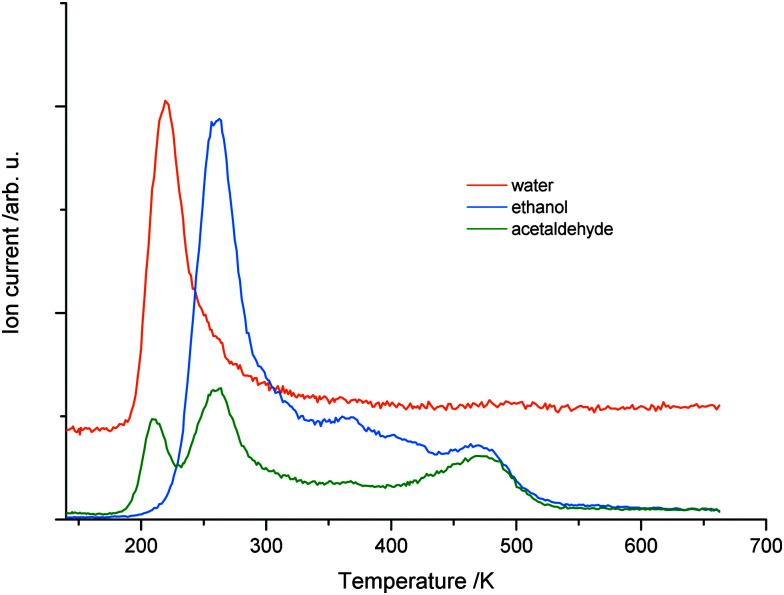
TPD after 1 min of UV excitation at 130 K for 1 L ethanol on a surface-oxidized crystal. On this surface the desorption features of the monitored species are observed at slightly higher temperatures. After the photoreaction, the production of acetaldehyde is found again. The water trace, however, shows only a more intense feature at around 225 K and no high temperature signal occurs.

To elucidate the role of defects in the reaction in more detail, a surface defect-free crystal is produced by annealing the crystal in oxygen at 300 K.^35,36^ The absence of any photon stimulated desorption (PSD) of O_2_ indicated that no O_2_-species is bonded to remaining defect sites.^2,35,37^ The resulting TPD after 1 min of photoexcitation (Fig. 5) shows a slight shift of 30 K to higher temperatures for the ethanol desorption in comparison to the desorption from a defect-rich TiO_2_(110) surface. A similar behavior is observed for the desorption of acetaldehyde, which is in good agreement with the literature for molecular acetaldehyde.^13^ In contrast to the defect-rich surface, only the H_2_O desorbing from bridging O-atoms is observed. Although the photoreaction of ethanol takes place, a higher temperature signal of H_2_O as on the defect rich surface is not observed. This further demonstrates that the surface of the crystal is indeed defect free. The integral for the peak of 210 K significantly exceeds the one in Fig. 4a, which shows that water is again formed during the photoreaction. In addition, the total amount of H_2_O desorbing from the surface after the same time of illumination is similar for both surfaces, the defect-free and the defect-rich ones.

## Discussion

### Defect rich surface

**Fig. 6:**
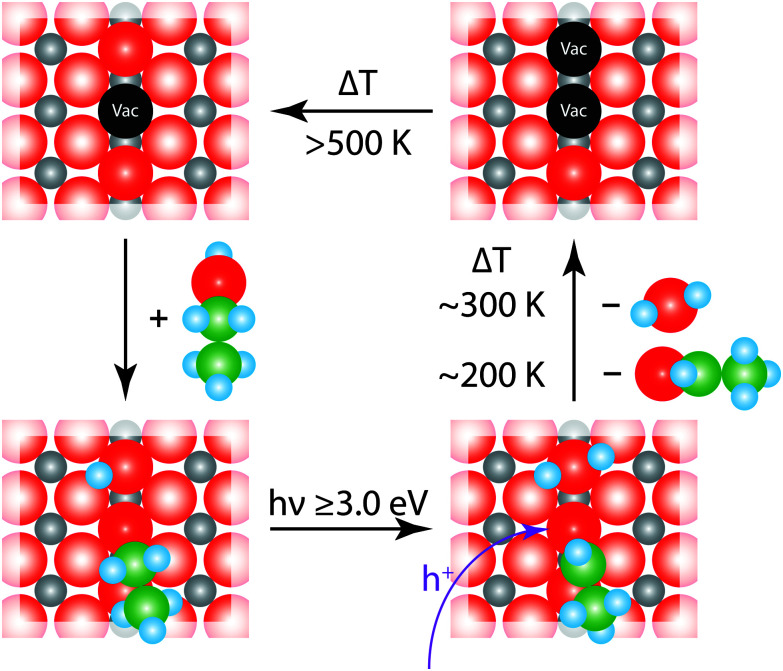
Photochemical reaction mechanism of ethanol on a defect-rich TiO_2_(110) surface. The figure illustrates four different reaction steps. After the dissociative ethanol adsorption, an ethoxy and a neighboring surface hydroxyl are formed. Under UV-illumination, charge carriers are generated in the semiconductor and the photoholes travel to the surface. There they react with ethanol under αH-abstraction to give acetaldehyde and water; the latter is in equilibrium with two surface hydroxyl groups. When the crystal is heated to elevated temperatures, the reaction products are desorbing at the corresponding temperatures and formally two oxygen vacancies remain. The surface is eventually reoxidized by bulk diffusion leading to a similar surface-defect density as before the photoreaction. (The grey balls resemble the Ti atoms and the red ones O atoms. The intense colored red balls indicate bridging oxygen atoms in the rows of the TiO_2_(110) surface. Shown in black are vacancies in those bridging atoms, also referred to as surface defect. Green balls are associated with C atoms and light blue resemble H atoms.)

Based on our findings and the existing studies in the literature, we propose the mechanism illustrated in Fig. 6 for the photochemical reaction on rutile. It is widely accepted that ethanol adsorbs preferentially in a dissociative way on rutile TiO_2_(110) and always dissociatively in an oxygen vacancy.^22,38,39^ Thus, in the defect a chemical chemical reaction according to eqn (1) takes place, which results in the formation of an ethoxy species and a protonated O_br_.

1 CH_3_CH_2_OH + O_br_ + Ti_vac_ → CH_3_CH_2_O–Ti_vac_ + OH_br_

When the semiconductor is irradiated by photons with energies above the band gap electron hole pairs are created (eqn (2)).

2 TiO_2_ + *hν* → TiO_2_ + e^−^ + h^+^

Depending on the type of photochemical reaction, either electrons or holes govern the yield in the surface chemistry.^8,37,40,41^ The oxidation of alcohols is a hole mediated process.^10,12,16,42^ It can be described by eqn (3), in which a formal h^+^ reacts with the partly negatively charged ethoxy species, which undergoes an α-H elimination.

3 CH_3_CH_2_O–Ti_vac_ + OH_br_ + h^+^ → CH_3_CHO_ad_ + H_2_O_ad_ + 2Ti_vac_

The resulting acetaldehyde is still bound on the surface *via* the oxygen atom,^43^ and can desorb at 195 K. The abstracted hydrogen either moves to the neighboring hydroxyl group to form water or results in the formation of another intermediate hydroxyl species, which eventually reacts to water and a bridging oxygen atom.^44^ In both cases, water molecules in an oxygen vacancy are produced, which block the defect and deactivate the catalyst. This mechanism fully supports the observed surface chemistry. The amount of water desorbing from defect sites shows a similar behavior to the formation of acetaldehyde (Fig. 2c and 4c). The reaction saturates, when all the surface defects are blocked by the reaction products. This is supported by studies for alcohols, where no diffusion effects have been reported at cryogenic temperatures below 200 K.^45,46^ In addition, it is observed that the amount for acetaldehyde saturation lies in the range of the density of defect states on the surface. At a temperature around 200 K the aldehyde is leaving the surface site. However, water molecules, which are in equilibrium with two hydroxyl species,^47^ still reside in the defects and cause a deactivation of the catalyst. This deactivation is observed after the consecutive dosing of ethanol after the photoreaction. If the water is eventually thermally removed above 450 K, the trap sites are accessible again. In addition, it is also well known that the surface is reoxidized from the bulk at these temperatures, so that the same percentage of surface defects is reobtained.^30,48^ Site blocking of the oxygen vacancies by the water molecule formed is also indicated by the absence of PSD of O_2_ even after the thermal removal of ethanol and acetaldehyde and the subsequent dosage of oxygen at low temperatures. Furthermore, it should be noted that no coking is observed, as the Auger spectra did not reveal transitions for carbon, even for temperatures of up to 800 K.

The role of defects in the photochemical reaction is also in good agreement with the fact that the defects such as oxygen vacancies lead to a stabilization of negative charges at the surface, which further results in an upward band bending in the semiconductor.^41,42^ This causes a preferential movement of photoholes to the defects, while the electrons travel into the bulk.^41^

Similar reaction and saturation behavior has been reported previously for methanol^16^ and 2-propanol^10^ and are a strong indication that the proposed mechanism may play an important role even in the photochemistry of other alcohols.

### Oxidized surface

Fig. 5 demonstrates that the photooxidation of ethanol can also be performed at the surface of an oxidized crystal. Due to the absence of oxygen vacancies, which is also reflected in the absence of any water desorption between 290 K and 420 K, another reaction pathway must be responsible for the acetaldehyde formation on this surface. In this respect, it is well established that dosing of O_2_ at 300 K results in the formation of dissociated O_ad_-atoms, which fill the vacancies and populate the surface.^36,48,49^

4 TiO_2_ + *hν* → TiO_2_ + e^−^ + h^+^

5 CH_3_CH_2_O_ad_ + OH_ad_ → CH_3_CHO + H_2_O_ad_

Instead of filling vacancies, ethanol adsorbs on the oxide surface and dissociates, resulting in the ethoxy species and an adsorbed hydroxyl species. After the light-induced creation of electron hole pairs, photoholes initiate the formation of acetaldehyde. The reaction takes place *via* the abstraction of an α-hydrogen atom of the ethanol and water is formed at the surface, eqn (5). This is supported by the increase in the water desorption at around 225 K. A similar desorption temperature is found for water that is bound molecularly to a bridging oxygen atom.^34^

## Conclusion

In summary, we propose a stoichiometric reaction mechanism for the illumination-dependent photochemical oxidation of ethanol to acetaldehyde on TiO_2_(110) surfaces. The mechanism explicitly addresses the role of surface defects (such as oxygen vacancies) and the role of water as a by-product of the reaction. With this mechanism the experimental observations of the role of oxygen in surface preparation and deactivation as well as saturation of the reaction can be explained. This is facilitated by two different experimental methods. The first one uses TPD as a probe for the accumulation of photo-products at cold temperatures and the second one investigates the photoreaction at elevated temperatures above the desorption temperature of the photo-product. Our mechanism can not only be extended to other alcohols of different chemical structures, but may even supply potential reaction pathways for metal particle loaded semiconductor systems.
